# Salmon Erythrocytes Sequester Active Virus Particles in Infectious Salmon Anaemia

**DOI:** 10.3390/v14020310

**Published:** 2022-02-02

**Authors:** Johanna Hol Fosse, Maria Aamelfot, Tonje Sønstevold, Simon Chioma Weli, Niccolò Vendramin, Petra Elisabeth Petersen, Anita Solhaug, Marit Måsøy Amundsen, Inger Austrheim Heffernan, Argelia Cuenca, Debes Hammershaimb Christiansen, Knut Falk

**Affiliations:** 1 Norwegian Veterinary Institute, 1433 Ås, Norway; aamariaa@hotmail.com (M.A.); tonje@skogseid.no (T.S.); simon.weli@vetinst.no (S.C.W.); anita.solhaug@vetinst.no (A.S.); marit.masoy.amundsen@vetinst.no (M.M.A.); inger.austrheim@vetinst.no (I.A.H.); knut.falk@gmail.com (K.F.); 2 Unit for Fish and Shellfish Diseases, National Institute of Aquatic Resources, Technical University of Denmark, 2800 Kongens Lyngby, Denmark; niven@aqua.dtu.dk (N.V.); arcun@aqua.dtu.dk (A.C.); 3 National Reference Laboratory for Fish and Animal Diseases, Faroese Food and Veterinary Authority, 110 Tórshavn, Faroe Islands; pep@hfs.fo (P.E.P.); debesc@hfs.fo (D.H.C.)

**Keywords:** orthomyxovirus, isavirus, red blood cell, adsorption, decoy, viral replication, nucleated erythrocyte

## Abstract

Infectious salmon anaemia virus (ISAV) binds circulating Atlantic salmon erythrocytes, but the relevance of this interaction for the course of infection and development of disease remains unclear. We here characterise ISAV-erythrocyte interactions in experimentally infected Atlantic salmon and show that ISAV-binding to erythrocytes is common and precedes the development of disease. Viral RNA and infective particles were enriched in the cellular fraction of blood. While erythrocyte-associated ISAV remained infectious, erythrocytes dose-dependently limited the infection of cultured cells. Surprisingly, immunostaining of blood smears revealed expression of ISAV proteins in a small fraction of erythrocytes in one of the examined trials, confirming that ISAV can be internalised in this cell type and engage the cellular machinery in transcription and translation. However, viral protein expression in erythrocytes was rare and not required for development of disease and mortality. Furthermore, active transcription of ISAV mRNA was higher in tissues than in blood, supporting the assumption that ISAV replication predominantly takes place in endothelial cells. In conclusion, Atlantic salmon erythrocytes bind ISAV and sequester infective virus particles during infection, but do not appear to significantly contribute to ISAV replication. We discuss the implications of our findings for infection dynamics and pathogenesis of infectious salmon anaemia.

## 1. Introduction

*Infectious salmon anaemia virus* (ISAV) is a segmented negative-stranded RNA virus that belongs to the *Orthomyxoviridae* family. ISAV exists in two variants. The first is a non-pathogenic variant that only appears to infect the mucosal epithelium [1]. This variant is referred to as HPR0, based on the presence of a full-length highly polymorphic region of segment 6. The commonly accepted hypothesis is that HPR0 represents the wild type form of the virus, from which pathogenic ISAV arises [1,2]. The pathogenic variant is referred to as HPR∆, based on deletions in the highly polymorphic region of segment 6 [3]. In contrast to HPR0, HPR∆ has acquired the ability to cause a generalized infection of vascular endothelial cells and may lead to severe disease in farmed Atlantic salmon, *Salmo salar* L. [3]. HPR∆ poses a serious economic and welfare concern to the global salmon aquaculture industry, and infection with either form of ISAV is notifiable to the OIE/World Organisation for Animal Health [4], whereas the European Union only requires notification of infection with HPR∆. For the rest of this article, the term ISAV will refer to the HPR∆ variant, unless otherwise noted.

The pathogenesis of infectious salmon anaemia (ISA) is characterised by severe anaemia and circulatory disturbances. In the late stages of disease, fish commonly present with pale gills and haematocrit values below 10% [3,5]. Other typical findings in diseased fish include ascites, multi-organ petechial haemorrhages, and congestion of the liver, kidney, and/or spleen [3,5,6]. The mechanism by which ISAV infection causes anaemia has not been characterised. One hypothesis is that the binding of ISAV to erythrocytes shortens their life span and contributes to the typical regenerative anaemia of late-stage infection [3]. ISAV attaches to cell surfaces by the ISAV haemagglutinin esterase (HE), a viral surface protein that binds 4-*O*-acetylated sialic acids. This sialic acid variant is expressed by epithelial cells, vascular endothelium, and erythrocytes [7,8,9]. Following an initial replication phase in mucosal epithelium, ISAV replicates in and buds from vascular endothelial cells [8,10,11]. Viral budding from the endothelium appears to be predominantly luminal [8,11], suggesting that most endothelial-produced viral particles enter the blood stream. In line with this, viraemia has been reported in several infection trials [2,12,13]. 

Many questions remain unanswered regarding how ISAV-erythrocyte interactions influence the course of infection and the development of ISA. Like several other viruses that target sialic acids, ISAV crosslinks erythrocytes in vitro in a reaction referred to as haemagglutination [14]. Over a course of several hours, most haemagglutinating viruses cleave their cellular receptor, allowing the agglutinated erythrocytes to elute from the reaction. Like these viruses, ISAV HE has a catalytic pocket with esterase activity distant from its receptor binding domain [9,15,16]. Accordingly, ISAV-agglutinated erythrocytes from rainbow trout successfully elute from ISAV-mediated agglutination [14,17]. Curiously, Atlantic salmon erythrocytes do not [14,17]. We do not currently know if this in vitro finding also translates to a difference in how robustly ISAV associates with circulating erythrocytes in infected fish of these species. However, extensive ISAV-coating of erythrocytes has been observed in experimentally infected Atlantic salmon [8]. This suggests that ISAV remains associated with erythrocytes during viraemia, at least for some time. The consequences of such binding are poorly understood. While no evidence of causality exists, the ability to elute from erythrocytes appears to relate inversely to the susceptibility to disease. Rainbow trout can sustain ISAV infection [18,19], yet experimental infection does not appear to induce signs of ISA in this species, with the exception of a single study where ISA-like disease was observed in a few family groups [19,20,21]. Furthermore, the one reported case of natural ISAV infection in rainbow trout was not associated with disease [18]. In contrast, Atlantic salmon is the only species where natural outbreaks of ISA have been reported.

Additionally, while one research group reported ISAV endocytosis [22] and replication [23] in virus-agglutinated erythrocytes, these findings have not been confirmed. Moreover, the studies have some limitations, including an uncertain relevance to the situation in infected fish and lack of measures to exclude non-erythrocytes from the inoculated cell populations. Altogether, it remains unknown if circulating erythrocytes are permissive to ISAV replication.

In this study, we analysed Atlantic salmon blood samples obtained from two independent ISAV infection trials with different outcomes, substantiating that viraemia and erythrocyte binding is a consistent and persisting feature of infection, and that blood-borne ISAV predominantly remains within the cellular compartment. A fraction of erythrocytes expressed ISAV proteins in one of the trials. However, such expression was rare and not required for disease and mortality to develop. Furthermore, ISAV mRNA transcription in blood was negligible, compared to that in heart and kidney. Therefore, despite a small subset of Atlantic salmon erythrocytes being at least partly permissive to ISAV infection, erythrocytes do not appear to contribute significantly to ISAV replication. Erythrocyte-bound ISAV remained infectious, yet erythrocytes inhibited serial infection of cultured cells in a decoy-like manner. Our findings raise the question of how viral-erythrocyte interactions modulate infection dynamics and disease pathogenesis in ISA.

## 2. Materials and Methods

### 2.1. Fish and Experimental Infection

The 2018 infection trial was recently described [24]. Briefly, Atlantic salmon postsmolts (mean weight 80 g, Stofnfiskur, Benchmark Genetics, Iceland) were infected with ISAV Glesvaer/2/90 by a 2-h immersion challenge (2 × 10^4^ TCID50/mL) and maintained in tanks supplied with filtered, UV-treated seawater (33‰ salinity). In the 2020 trial, Atlantic salmon presmolts (mean weight 60 g, Stofnfiskur, Benchmark Genetics, Iceland) were infected with ISAV Glesvaer/2/90 by a 2-h immersion challenge (10^3.5^ TCID50/mL, which translates to a 6-fold lower virus concentration than the 2018 trial) and maintained in 180-litre round tanks supplied with fresh water. Both trials used unvaccinated fish that had been tested and found negative for the presence of common infectious agents, including ISAV, infectious pancreatic necrosis virus, piscine myocarditis virus, piscine orthoreovirus, and salmon gill poxvirus. In addition, fish in the 2018 trial were tested and found negative for salmonid alphavirus, and fish in the 2020 trial were tested and found negative for piscirickettsia salmonis, viral haemorrhagic septicaemia virus, and infectious haematopoietic necrosis virus. The water temperature in both trials was 12 °C. Blood for in vitro experiments was collected from Atlantic salmon (weights between 100–200 g, Atlantic QTL InnOVA SHIELD/RED, AquaGen, Trondheim, Norway) maintained in fresh water, 12–14 °C. All blood sampling was performed on deeply anaesthetised fish. Anaesthesia was induced by immersion in water containing 100 mg/mL tricaine methanesulfonate (MS-222 or Tricaine Pharmaq, Pharmaq, Overhalla, Norway), and blood was collected from the caudal vein in heparinised tubes. After blood sampling, fish were examined for gross pathological changes and killed by cervical sectioning. Organs were harvested into RNAlater (#AM7021, Thermo Fisher Scientific, Waltham, MA, USA) for nucleic acid analyses and 10% buffered formalin for histopathology and immunohistochemistry. Haematocrits were measured within 1–2 h after blood sampling.

### 2.2. Evaluation of Erythrocyte Osmotic Fragility

Erythrocyte fragility was evaluated as previously described [25], with some minor modifications. Briefly, blood was diluted 1:1 in phosphate-buffered saline (PBS), 10 µL of this suspension was added to 500 µL aliquots of serially diluted NaCl (concentration 3–10 g/L), mixed gently by inversion, incubated for 20 min at room temperature, and centrifuged (800× *g*, 4 min, room temperature). Supernatants were transferred to 96-well plates, and haemolysis was estimated by measuring the optical density at 405 nm by spectrophotometry (Multiskan^TM^ SkyHigh, Thermo Fisher Scientific).

### 2.3. Cells

Atlantic salmon erythrocytes (red blood cells, RBC) were purified from heparinised blood diluted 1:2–1:10 in PBS, layered on a 51% Percoll Plus (#17-554-01, GE Healthcare Life Sciences, Chicago, IL, USA) gradient, and centrifuged (400× *g*, 30 min, 4 °C). When used for in vitro culture, RBC were washed (3 × PBS) and resuspended (2.0 × 10^7^ cells/mL) in L-15 medium (#12-700F, Lonza, Basel, Switzerland) supplemented with 1% Penicillin/Streptomycin/Amphotericin (#17-745E, Lonza), 10% FBS (Fetal Bovine Serum, #DE14-801F, Lonza), and 2% L-glutamine (#17-605E, Lonza), hereafter referred to as culture medium. The cell suspension was transferred to 6-well cell culture plates (3 mL, i.e., 6.0 × 10^7^ cells/well) and incubated on a digital 2/4 microplate shaker (#0003208000, IKA-Werke GmbH & Co. KG, Staufen, Germany) at 15 °C. Atlantic salmon kidney (ASK [20]) cells were maintained in the same culture medium as the RBC, kept at 20–23 °C, split 1:3 every other week, and used between passage 45 and 55.

### 2.4. Viruses and Generation of Infective Material

The Norwegian ISAV isolate Glesvaer/2/90 [26] (NCBI GenBank accession numbers HQ259671.1-HQ259678.1) was used throughout this study. ISAV was propagated in ASK cells as previously described [8,24]. Briefly, cellular monolayers were inoculated with ISAV in FBS-free culture medium for 4 h at 15 °C before addition of culture medium with 2% FBS and incubation at 15 ° C. Virus supernatants were harvested and cleared by filtration (0.2 µm) or centrifugation (3800× *g*, 10 min, 4 °C). Virus stocks were stored at −80 °C. Infective titres were calculated by the 50% tissue culture infective dose (TCID50/mL) as previously described [14]. Membrane-fractions of infected ASK cells for use in the virus binding assay were collected as described elsewhere [8]. Briefly, cells were washed (3 × PBS), detached by a cell scraper, and pelleted by centrifugation (500× *g*, 10 min, 4 °C). Pellets were washed (2 × PBS), lysed by three cycles of freeze-thawing, and cleared supernatants containing cell membrane fractions with high expression levels of ISAV surface proteins were collected after centrifugation (10,000× *g*, 10 min, 4 °C). Haemagglutination titres of supernatants were determined using RBC harvested from non-infected Atlantic salmon, suspended in PBS (0.5%, i.e., 1.0 × 10^7^ cells/mL).

### 2.5. Preparation of RBC Membrane Fractions

RBC membrane fractions were prepared based on a previously described protocol [27] with some modifications. Briefly, RBC from two individual Atlantic salmon were mixed 1:1, and 100 µL of this cell suspension was lysed by 1:10 dilution in ice cold water with 1% protease inhibitors (#P8340, Sigma-Aldrich, Merck KGaA, Burlington, MA, USA, 10 min on ice). The cells were homogenized with a tight-fitting dounce (20 strokes). After addition of 1000 µL of buffer A (12.5 mM MgCl, 15 mM EDTA, 75 mM Tris, pH 7.5), the solution was homogenized again. To remove nuclei and organelles, the cell suspension was centrifuged (5000× *g*, 5 min), and the supernatant was transferred to an ultracentrifuge tube, placed on ice. The homogenisation procedure was repeated three times with the cell pellet in buffer A diluted 1:1 with water. The pooled supernatant fractions were then centrifuged (40,000× *g*, 30 min) and the membranous pellet resuspended in 25 µL buffer B (20 mM Tris, 2 mM EDTA, pH 7.5) and stored at −80 °C.

### 2.6. Blotting, Virus Binding Assay, and Staining of Erythrocyte Membrane Fractions

SDS-PAGE and Western blotting (NuPage Novex system, Invitrogen, Thermo Fisher Scientific) was performed using 10 µL of the RBC membrane lysate. Three parallel 0.45 µm nitrocellulose membranes (#162-0115, BioRad laboratories, Hercules, CA, USA) were prepared in each experiment. One membrane was used for periodic acid schiff (PAS) staining for glycoproteins as described by the manufacturer (Pierce glycoprotein staining kit, #24562, Thermo Fisher Scientific). The other two membranes were used for the virus binding assay: one membrane was saponified (30 min, 0.1 m NaOH, 37 °C) prior to the assay to remove acetylations. The membranes were blocked in 3% dry milk in tris-buffered saline with 0.05% Tweens (TBST, 60 min, room temperature), washed (2 × 10 min, TBST), incubated with membrane fractions of ISAV-infected ASK cells (512 haemagglutinating units/mL, 1 h, room temperature), as described elsewhere [8], washed (3 × 15 min, TBST), incubated with mouse IgG_1_ specific to ISAV HE (clone 3H6F8, 1:150, 1 h, room temperature), washed (3 × 15 min, TBST), incubated with HRP-conjugated horse anti-mouse IgG (#7076, Cell Signaling, Danvers, MA, USA, 1:1000, 1 h, room temperature), and washed again (3 × 15 min, TBST). Virus binding was detected by chemiluminescence, using Super Signal West pico plus substrate (#34579, Thermo scientific) and Chemidox XRS+ (BioRad laboratories). Gels were stained for total protein by SimplyBlue SafeStain (#LC6060, Invitrogen).

### 2.7. Experimental Infection of Cells

For inoculation of plasma and RBC from infected fish, ASK cells were seeded in flat-bottom 96-well culture plates and allowed to reach confluence. The inoculates were added to plates in 5-fold dilutions, starting at 10 µL per well, and plates were incubated at 15 °C for 5 days before fixation in 80% acetone (Sigma-Aldrich) and immunofluorescent staining for ISAV, as described in Section 2.8. Infective titres were calculated by the 50% tissue culture infective dose (TCID50/mL) as previously described [14]. For visualisation of viral proteins in ASK cells, cells were seeded in 8-well µ-slides (#80826, Ibidi GmbH, Gräfelfing, Germany), inoculated 24 h, infected, and fixed in 4% paraformaldehyde (Sigma-Aldrich) at the given time points. For RBC/ASK co-culture, ASK cells were seeded in flat bottom 96-well culture plates (3.0 × 10^3^/well), incubated at 20 °C for 24 h, infected with ISAV (1.0 × 10^3^ TCID50/well), and incubated at 15 °C for 24 h. Next, a two-fold serial dilution of RBC (1:2 to 1:256) was made from a 10^6^ RBC/mL suspension, and 25 µL added per well, exposing infected ASK cells to a range of 1 × 10^2^–1.25 × 10^4^ RBC/well. All samples were incubated on a digital 2/4 microplate shaker at 15 °C for 48 h. Following incubation, supernatants from each well were collected and transferred onto uninfected ASK cultures in 96 well plates and incubated on a digital 2/4 microplate shaker at 15 °C for another 48 h. After incubation, the culture plates were washed (PBS), fixed in 80% acetone and air-dried before immunofluorescent staining for ISAV nucleoprotein, as described in Section 2.8. The number of green fluorescent cells was counted by using the Spectramax i3x plate reader, minimax 300 Imaging Cytometer module (Molecular devices, San Jose, CA, USA). The binding of released ISAV to inoculated RBC was simulated by incubating serial dilutions of RBC as described above with ISAV 1 × 10^3^ TCID50/mL on the 2/4 digital microplate shaker (60 min, 15 °C), washing the RBC in PBS, and lysing them in RLT buffer (#79216, Qiagen, Hilden, Germany) before RNA extraction and qPCR as described in Section 2.9.

### 2.8. Immunostaining and Microscopy

For immunofluorescent staining of acetone-fixed blood smears and acetone- or paraformaldehyde-fixed cells for viral proteins, the following antibodies were used: mouse IgG_1_ targeting ISAV HE (clone 3H6F8 [28], 1/100) and ISAV nucleoprotein (#P10, Aquatic Diagnostics Ltd., Stirling, Scotland, 1/500) and/or rabbit polyclonal serum reactive with recombinant ISAV matrix protein (K806, 1/50). Goat anti-mouse IgG or IgG/IgM–Alexa 488 (#A11001 or #A10680, Molecular Probes, Thermo Fisher Scientific, used 1/400 and 1/200) and goat anti-rabbit IgG–Alexa 594 (#A11012, Molecular Probes) were used for detecting bound antibodies. Briefly, blood smears were incubated with 5% dry milk in PBS (30 min, room temperature) before incubation with primary antibody diluted in blocking buffer (60 min, room temperature), washed (3 × 5 min PBS), incubated with secondary antibody diluted in blocking buffer (45 min, room temperature), washed (2 × 5 min PBS, 1 × 5 min PBS with Hoechst 33,342 [Molecular Probes, 1:5000], 1 × PBS), air dried and mounted in ProLong Gold Antifade Mountant (# 10144, Molecular Probes). The same protocol was used for staining cells, but blocking was omitted, and antibodies were diluted in PBS only. Wide-field fluorescent microscopy was performed using a Zeiss Axiocam 503 equipped with a N-Achroplan 63×/0.85 Ph3 M27 objective. Confocal microscopy was performed using a Zeiss LSM 710 equipped with a Plan-Apochromat 63×/1.40 oil DICM27 objective. Uncropped micrographs are included as Supporting information. Original image files can be obtained upon request.

For immunohistochemistry, sections of formalin-fixed paraffin-embedded tissues were placed on Superfrost slides, deparaffinised, rehydrated, and heat treated (60–70 °C, 20 min). Immunohistochemistry was performed as previously described [8]. Antigen retrieval was performed by incubation in citric acid (0.1 M, pH 6.0) for 5 min in the microwave (800 W), followed by cooling in the retrieval buffer for another 15 min. Sections were washed (TBS), incubated with blocking buffer (2% goat serum, 5% dry milk, 20 min), incubated with rabbit anti-ISAV nucleoprotein (K716, 1:3000, 4 °C, overnight), washed (TBS), and signal was visualised by the Vectastain ABC anti-rabbit IgG AP Immunodetection kit (#AK-5001, Vector laboratories, Burlingame, CA, USA), according to manufacturer’s instructions. Sections were counterstained by haematoxylin, mounted, and evaluated by light microscopy, using a Leica DM5000B. 

### 2.9. RNA Extraction and qPCR 

Three laboratories took part in RNA extractions and qPCR analyses, performed according to each laboratory’s established protocols.

Starting materials for qPCR analyses included full blood (both trials) density gradient-purified RBC (2020 trial, 20 μL), plasma (2020 trial, 50 μL), head kidney (2020 trial), heart (2020 trial), sucrose-purified ISAV, and in vitro cultured ASK cells (confluent wells, 6-well plate) and RBC (10^7^ cells for measuring ISAV replication and the response to infection; RBC pellets of reducing size, according to dilution, for measuring decoy function). Full blood from the 2020 trial was lysed and homogenized using the Indimag pathogen kit (#SP947457, Indical biosciences, Leipzig, Germany) on the IndiMag automated platform (Indical bioscience). Remaining samples were lysed in RLT buffer (#79216, Qiagen). In vitro cultured ASK and RBC were homogenized using QIA-shredder spin column (#79656, Qiagen) for 2 min at maximum speed. Blood, plasma, and tissue samples in RLT were homogenized by 3–5 mm steel beads in a TissueLyser II (#85300, Qiagen, 24.7–30 Hz, 2 × 4–5 min).

RNA was extracted by the RNeasy Mini kit (#74106, Qiagen) (Samples for comparison of virus levels in blood at the peak of infection in the two trials; description of infection dynamics and viral transcription in full blood in the 2018 trial; RBC in vitro experiments), the IndiMag Pathogen kit on the IndiMag automated platform (Indical bioscience) (Samples for description of infection dynamics in the 2020 trial), or by the QIAsymphony RNA kit (#931636, Qiagen) on the QIAsymphony automated platform (Qiagen) (Samples for comparison of virus levels in blood cells and plasma; comparison of viral transcription in blood and tissues in the 2020 trial). After RNA extraction, a NanoDrop™ 2000 spectrophotometer (Thermo Fisher Scientific) was used to estimate purity and yield of RNA, and samples were stored at −80 °C. 

One-step qPCR (Samples for description of infection dynamics in full blood in the 2020 trial; comparison of virus levels in blood cells and plasma; comparison of viral transcription in blood and tissues in the 2020 trial) was performed either on the Stratagene MX300P instrument (Agilent) (Samples for description of infection dynamics in the 2020 trial) with 5 μL input RNA, TaqPath 1-step RT-qPCR mastermix, CG (Thermo Fisher Scientific), 900 nM of each primer, and 250 nM probe in a 20 μL reaction volume, or on the QuantStudio 5 real-time PCR instrument (Thermo Fisher Scientific) (Samples for comparison of virus levels in blood cells and plasma; comparison of viral transcription in blood and organs in the 2020 trial) with 2 μL input RNA, TaqPath 1-Step RT-qPCR Master Mix (Thermo Fisher Scientific), 1 μM of each primer, and 200 nM probe in a 10 μL reaction volume.

For experiments based on 2-step qPCR (Samples for comparison of virus levels in blood at the peak of infection in the two trials; description of infection dynamics and viral transcription in full blood in the 2018 trial; RBC in vitro experiments), cDNA was synthesised by the QuantiTect Reverse Transcription kit (#205311, Qiagen) with gDNA elimination, according to manufacturer’s instructions. 0.5–1 ug RNA in a 20 μL total reaction volume was used for cDNA synthesis, except in the experiment that measured how much ISAV was sequestered in a RBC pellet of diminishing size, rather than the ISAV sequestered per cell. To address this, RNA input was normalised according to volume. cDNA samples were stored at −20 °C until 2-step qPCR was performed, using the CFX384 Touch Real-Time PCR Detection System (BioRad laboratories). Samples from in vitro cultured ASK cells and RBC were analysed in a SYBR Green assay using 2 µL (5 µg) input cDNA, 2x SsoAdvanced Universal SYBR Green Supermix (#1725270, BioRad laboratories), and 10 µM of each primer in a 10 μL reaction volume. Blood samples (Samples for comparison of virus levels in blood at the peak of infection in the two trials; description of infection dynamics and viral transcription in full blood in the 2018 trial) were analysed in TaqMan assays, using 2 µL (5 µg) input cDNA, 2× TaqMan Fast Advanced Master Mix (#4444556, Thermo Fisher Scientific), 10 µM of each primer, and 10 µM probe in a 10 μL reaction volume. Data were analysed using the CFX Manager software (version 3.1.1621.0826, BioRad laboratories).

Standard curves of synthetic DNA fragments (gBlocks) containing the relevant target sequences (Integrated DNA Technologies, Coralwille, IA, USA) were used for calculation of copy number per µg RNA or µL sample, as relevant. Sequences for primer/probe sets and gBlocks are provided in Supplementary Tables S1 and S2, respectively.

### 2.10. Statistics

Graph Pad Prism 9 for Windows 64-bit (version 9.0.1) was used for visualisation of data and statistics. Descriptive data were extracted from column statistics. Where appropriate, significance testing of non-parametric data was performed by the Mann–Whitney U, Kruskall–Wallis, or Wilcoxon matched-pairs signed ranks tests. Significance testing of parametric data was performed by one sample t test and the RM 2-way ANOVA with the Geisser Greenhouse correction.

## 3. Results

### 3.1. ISAV Binds Erythrocyte Membrane Glycoproteins

**Figure 1:**
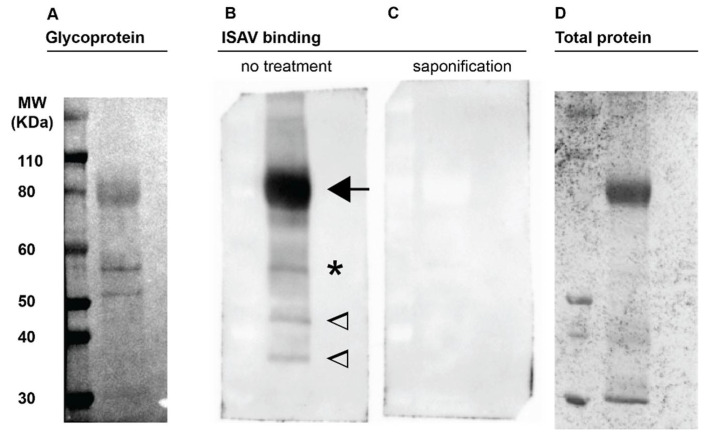
ISAV binds Atlantic salmon erythrocyte membrane glycoproteins. Plasma membrane-enriched erythrocyte lysates were separated by gel electrophoresis under denaturing conditions and blotted to nitrocellulose membranes. (**A**) Periodic acid-schiff staining to visualise glycoproteins. The numbering to the left indicates the weight of the molecular marker in the left lane. (**B**,**C**) Virus binding assay to blotted membranes from parallel runs, visualising bound antigen by detection of ISAV HE. (**B**) Arrow points to the predominant ISAV-binding glycoprotein-containing band; star indicates a second ISAV-binding glycoprotein-containing band; in contrast, two ISAV-binding bands at 35 and 45 kDa indicated by open arrowheads, did not have obvious corresponding PAS bands. (**C**) Prior saponification of the blotted membrane obliterated all ISAV binding. (**D**) Total protein staining of representative gel. Images show full-length lanes representative of three independent experiments. Original images have been uploaded as supporting information.

ISAV attaches to cellular 4-*O*-acetylated sialic acids [9,15]. Detection of glycoproteins in Atlantic salmon erythrocyte membrane fractions by periodic acid-schiff (PAS) staining revealed three clear glycoprotein-containing bands, including a diffuse band at the level of the 80 kDa marker and two additional smaller bands located between the 50 and 60 kDa markers (Figure 1A). A virus binding assay (Figure 1B) revealed that ISAV bound the glycoprotein-rich band at the 80 kDa marker, the upper of the two glycoprotein-rich bands between the 50 and 60 kDa markers (Figure 1A,B, arrow and star), and two bands at the level of the 35 and 45 kDa molecular markers with no obvious accompanying PAS signal. The lower glycoprotein-containing band between the 50 and 60 kDa markers did not appear to bind ISAV. Saponification of membranes prior to the virus binding assay obliterated all ISAV-binding, presumably by removal of 4-*O*-acetyl-groups (Figure 1C).

### 3.2. In ISAV-Infected Fish, a Fraction of Erythrocytes Becomes Coated with Viral Particles, and the Erythrocyte Osmotic Fragility Increases

**Figure 2:**
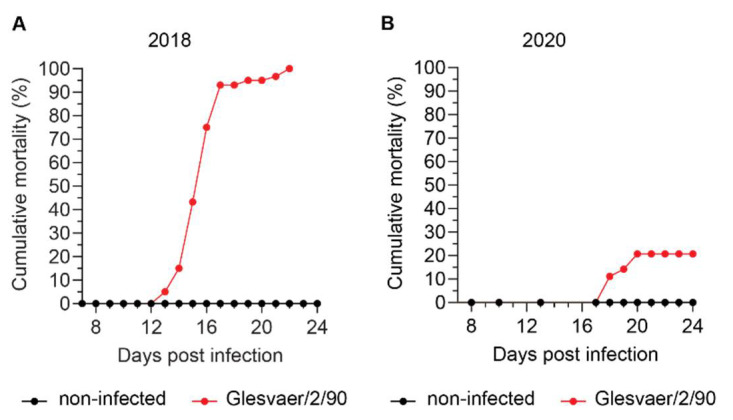
**Cumulative mortality in the two ISAV infection trials used in this study.** Materials analysed in this study were harvested from two independent ISAV infection trials conducted at different locations and in different years. (**A**) Cumulative mortality in the 2018 trial (results have been published previously [24], but are included for reference). (**B**) Cumulative mortality in the 2020 trial.

To better characterise interactions between ISAV and erythrocytes in infected Atlantic salmon, we analysed serial blood samples from two independent infection trials based on ISAV immersion challenge. The trials were performed at the Industrial and Aquatic Laboratory (Bergen, Norway) in 2018 and at the Danish National Institute of Aquatic Resources (Lyngby, Denmark) in 2020, respectively. Other aspects of the 2018 trial were recently reported in another publication [24]. The outcomes of infection were very different (Figure 2). As reported [24], infected fish in the 2018 trial started to die 12 days post infection (d.p.i.), with mortalities rapidly progressing to 100% at 21 d.p.i. In contrast, the 2020 trial resulted in less severe disease. No fish died before 18 d.p.i., and the cumulative mortality was 21% at 24 d.p.i. 

**Figure 3:**
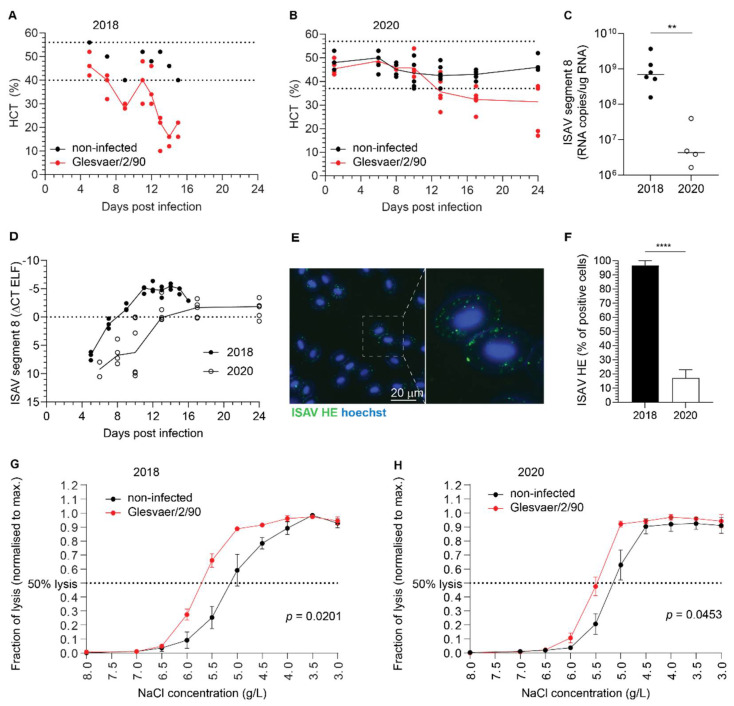
ISAV coats erythrocytes in experimentally infected Atlantic salmon. (**A**,**B**) A reduction in haematocrit was observed in both the 2018 (**A**) and 2020 (**B**) trials. Dots represent individual fish, solid lines connect median values, and dotted lines indicate the range of values in the non-infected fish group. (**C**) ISAV segment 8 qPCR in blood sampled at the peak of viraemia (12 + 13 d.p.i and 13 d.p.i., respectively) in the 2018 and 2020 trials. Dots represent individual fish, bars show median values. ** *p* = 0.0095, Mann–Whitney U. (**D**) Viral infection dynamics in full blood in the 2018 and 2020 trials. Dots represent individual fish, lines connect median values. (**E**) Wide-field micrograph of typical appearance of immunostaining for ISAV HE (green) in blood smear from infected fish (here: 5 d.p.i., 2018 trial). Nuclei are shown in blue. Image signal was enhanced in ImageJ [29] (version 2.1.0/1.53c; Java 1.8.0_172 [64-bit]) using the multiply function (process > maths > multiply). (**F**) The percentage of HE-positive erythrocytes at the peak of viraemia in both trials. Bars show median +/− 95% confidence intervals of manual counts from 10 microscope fields. **** *p* < 0.0001, Mann–Whitney U. (**G**,**H**) Osmotic fragility of erythrocytes in the infection trials. The graphs show mean erythrocyte lysis at each NaCl concentration +/− SEM of 3 (2018) or 5 (2020) individual infected fish harvested at 9 d.p.i (2018) or 13 d.p.i. (2020), compared to 8 (2018) or 5 (2020) non-infected controls. *p*-values give the significance of the difference between control and infected fish, as assessed by a RM 2-way ANOVA with the Geisser Greenhouse correction.

Haematocrit values were measured in both trials and defined as reduced if below the range observed in non-infected individuals (Figure 3A,B). In the 2018 trial (Figure 3A), the range of haematocrit values in non-infected fish (*n* = 8) was 40–56% (median 49%, interquartile range 42–52%). Reduced haematocrits were detected in 1/3 fish at 7 d.p.i., 3/3 fish at 9 d.p.i., 1/3 fish at 11 d.p.i., 2/3 fish at 12 d.p.i., 3/3 fish at 13 d.p.i., 3/3 fish at 14 d.p.i., and 3/3 fish at 15 d.p.i. Haematocrits were severely reduced, reaching 10% in individual fish. In the 2020 trial (Figure 3B), the range of haematocrit values in noninfected fish (*n* = 48) was 37–57% (median 46%, interquartile range 43–48%). Anaemia in the infected fish group was less pronounced and transient compared to the 2018 trial, with reduced haematocrits detected in 3/5 fish at 13 d.p.i., 4/5 fish at 17 d.p.i., and 2/5 fish at 24 d.p.i. Viraemia developed in both trials. Interestingly, and despite a difference in the peak viral load in blood (Figure 3C), the infection dynamics in full blood appeared similar, with viraemia peaking at 12–13 d.p.i. (Figure 3D). Immunostaining of blood smears from infected fish with an antibody targeting ISAV HE revealed prominent coating of erythrocytes with HE-positive particles in both trials. The appearance of a typical ISAV-coated erythrocyte is shown in Figure 3E. The coating of erythrocytes was most extensive in the 2018 trial (Figure 3F), in agreement with the blood viral loads. In both trials, ISAV-coating of erythrocytes preceded the development of anaemia and mortality, becoming obvious at 4 d.p.i. (2018 trial) and 13 d.p.i. (2020 trial), respectively. In line with early work that showed increased osmotic fragility in erythrocytes from severely anaemic fish from Norwegian ISA outbreaks in the late 1980s [25], we observed an increase in erythrocyte osmotic fragility in both infection trials that corresponded in time to the onset of anaemia (Figure 3G,H). The increase in osmotic fragility observed in our studies was less prominent than that previously reported from the outbreak situation.

### 3.3. ISAV Particles Are Enriched in the Erythrocyte Fraction of Blood

**Figure 4:**
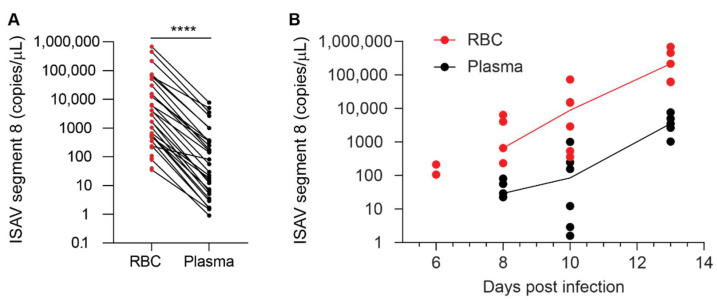
ISAV is enriched in the cellular fraction of blood. qPCR of density gradient-purified erythrocytes (RBC, shown in red) and plasma (black) from individual fish in the 2020 trial showing (**A**) the relationship between ISAV mRNA in RBC and plasma in all individual fish and (**B**) the relationship of ISAV mRNA in RBC and plasma over the time course of the experimental infection. Dots represent individual fish. (**A**) Lines connect values from the same fish. **** *p* < 0.0001, Wilcoxon matched-pairs signed rank test. (**B**) Lines connect median values within each group. The values on the *y*-axis have been adjusted for the difference in the sampled volume of RBC and plasma.

We next assessed ISAV in the different blood compartments of infected Atlantic salmon. To compare the proportion of virus that resided in the non-cellular and cellular fractions of blood, we measured viral RNA in plasma and density gradient-purified erythrocytes from the 2020 trial. RNA was extracted from homogenised samples containing 50 μL plasma or 20 μL erythrocytes and subjected to one-step qPCR as described in Materials and Methods. The number of viral copies per μL sample was normalised to the difference in sample input. We observed that virus levels in individual fish were always highest in the erythrocyte fraction of blood (Figure 4A). The levels of ISAV in erythrocytes remained higher than in plasma at all tested time points (Figure 4B).

### 3.4. On Rare Occasions, Circulating Salmon Erythrocytes Express ISAV Proteins, but This Is Not Required for Disease and Mortality

**Figure 5:**
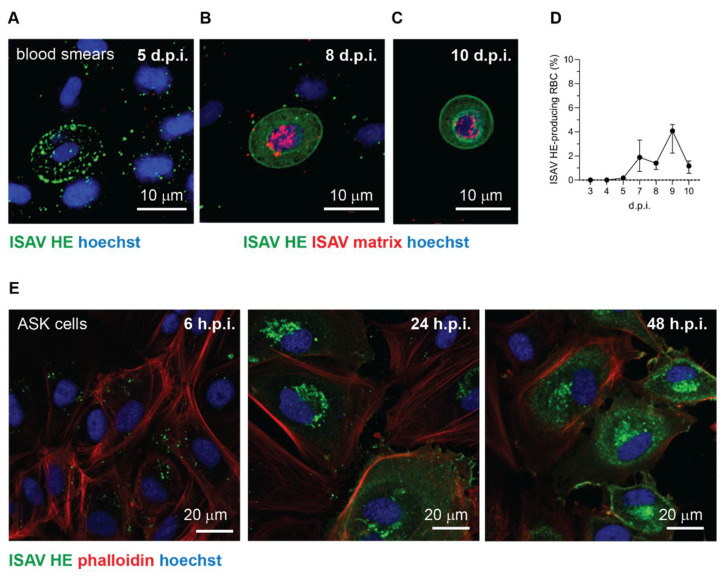
Occasionally, a subset of erythrocytes in infected Atlantic salmon express ISAV proteins. (**A**–**C**) Confocal micrographs of immunostained blood smears from ISAV-infected fish in the 2018 trial. (**A**) Example of the most frequently observed pattern with extensive coating of erythrocytes with HE-positive particles. (**B**,**C**) Examples of the few erythrocytes (**B**) and rare rounded erythrocyte-like cells (**C**) that express ISAV HE and matrix proteins. Images show maximum intensity projections from z-stacks. (**D**) Percentage of HE-expressing erythrocytes in the 2018 trial. The graph shows medians (dots) and 95% confidence intervals (bars) calculated from manual counts of 10 microscope fields, 3 fish per time point. (**E**) Confocal micrographs of immunostained ISAV-infected ASK cells illustrate the typical change in signal pattern as the infectious cycle progresses. In the initial stage of infection, staining reveals a bright punctuate pattern (left panel). Once cells start to express ISAV HE protein, a bright perinuclear signal appears in the region of the Golgi apparatus (middle panel). Finally, extensive cytoplasmic and membrane staining is present in the late stage of infection (right panel). Images in panel E were obtained in a single z plane.

Immunostaining of blood smears from infected fish showed that most erythrocytes that stained positive for ISAV HE were typical of cells in early-stage infection. These cells showed signal in bright granular foci, compatible with aggregates of viral particles on the cell surface and/or in endosomal compartments before fusion (Figure 5A). Nevertheless, the signal pattern in the 2018 trial was more diverse. In addition to the typical erythrocyte-coating with viral particles (Figure 5A), a small fraction of erythrocytes showed a homogenous signal pattern consistent with cytoplasmic and membrane localisation, compatible with cellular expression of HE (Figure 5B–D). The same cells also showed a positive nuclear signal for ISAV matrix protein (Figure 5B,C). A few of the ISAV protein-producing cells had a rounded morphology, consistent with an immature erythroid phenotype (Figure 5C). However, the nature of these rounded erythrocyte-like cells could not be verified, due to their rare occurrence. To illustrate the characteristic staining patterns that correspond to the stages of infection, we have included images of ISAV HE immunostainings in infected ASK cells (Figure 5E). The first hours after infection, the signal is bright and clearly demarcated (Figure 5E, left panel), similar to the signal observed in most HE-positive erythrocytes. Over the next 12–24 h, bright signal becomes evident in the Golgi apparatus, representing cellular HE synthesis (Figure 5E, middle panel). Finally, 24–48 h.p.i., cellularly expressed HE in the cytoplasm and at the cell membrane is clearly visible as homogeneous cellular signal with a brighter rim at the cell periphery (Figure 5E, right panel).

No evidence of viral protein expression in erythrocytes was observed in blood smears collected during the 2020 trial or in archive blood smears from 16 ISAV-infected fish (6–21 days post infection) from a previously reported trial that resulted in mild anaemia and 100% mortality [12,30] (data not shown). Moreover, stainings of blood smears made on-site in numerous field outbreaks with the same antibody used here, have shown a pattern consistent with Figure 5A, but not the patterns seen in Figure 5B,C [Knut Falk, personal observation]. Our collected findings suggest that ISAV protein expression in erythroid lineage cells is possible, but very rare, and not required for development of disease.

### 3.5. ISAV mRNA Is Predominantly Produced in Solid Organs

**Figure 6:**
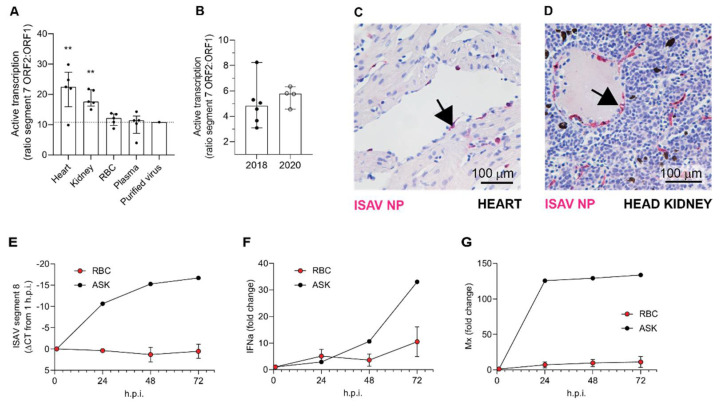
ISAV predominantly replicates in endothelial cells of solid organs. (**A**,**B**) Active viral transcription was estimated by calculating the ratio between ISAV segment 7 mRNA and RNA. Dots show values in individual fish, bars show median and interquartile ranges. (**A**) ISAV transcription in heart and kidney was higher than in RBC, plasma, and purified ISAV (13 d.p.i., 2020 trial). Stars indicate the difference between active transcription in RBC compared to heart (** *p* = 0.0087) and head kidney (** *p* = 0.0079), Mann–Whitney U. (**B**) ISAV transcription in full blood from fish harvested 12 and 13 d.p.i. in the 2018 trial (2018) and in erythrocytes harvested 13 d.p.i. in the 2020 trial (2020), detecting no significant difference between trials. (**C**,**D**) Micrographs showing immunostaining for ISAV nucleoprotein (NP, magenta) in formalin-fixed paraffin-embedded heart (**C**) and head kidney (**D**) from fish harvested 13 d.p.i. in the 2020 trial. Arrows point to ISAV NP-positive endothelial cells. Scalebars are 100 µm. (**E**–**G**) RBC from healthy fish were infected ex vivo with ISAV and harvested 1, 24, 48, and 72 h post infection (h.p.i.). qPCR was used to measure (**E**) ISAV segment 8, (**F**) IFNa, and (**G**) Mx transcripts. ELF-1α was used as reference for calculating fold change in F and G by the ΔΔCT method. The graphs show means +/− standard deviations from RBC from 4 individual fish. A representative time curve of results from infected ASK cells is included as reference.

To further investigate the role of the blood compartment in viral replication, we estimated viral mRNA transcription with the aid of a mRNA-specific qPCR that measures the ratio between spliced and unspliced transcripts from genomic segment 7 (ORF2 and ORF1, encoding the nuclear exporting protein and the nonstructural protein 1, respectively) [31]. We compared ISAV transcription in erythrocytes to that in heart and head kidney, both considered major sites for ISAV replication, and to plasma and sucrose-purified virus, where no viral transcription is expected. We observed that viral transcription in erythrocytes was similar to that in plasma and purified virus (Figure 6A). Moreover, the ratio between spliced and unspliced segment 7 transcripts in full blood 12 and 13 d.p.i. in the 2018 trial was similar to that in erythrocytes 13 d.p.i. in the 2020 trial, i.e., at the peak of viraemia (Figure 6B). In agreement with previous findings, the vascular endothelium strongly expressed ISAV nucleoprotein (NP) in both heart and kidney, as demonstrated by immunohistochemistry (Figure 6C,D). In agreement with our findings in infected Atlantic salmon, ISAV transcription in in vitro-infected salmon erythrocytes was negligible (Figure 6E). These results suggest that despite large amounts of viral particles being sequestered in the blood, viral replication predominantly occurs within endothelial cells of solid organs. As reported previously, in vitro-infected salmon erythrocytes upregulated the expression of interferon-alpha (IFNa; IFNa3-like, transcript variants X1 and X2) and the antiviral protein Mx (transcript variant X1), but to a much lesser extent than ASK cells (Figure 6F,G).

### 3.6. Erythrocyte-Sequestered ISAV Remains Infectious

**Figure 7:**
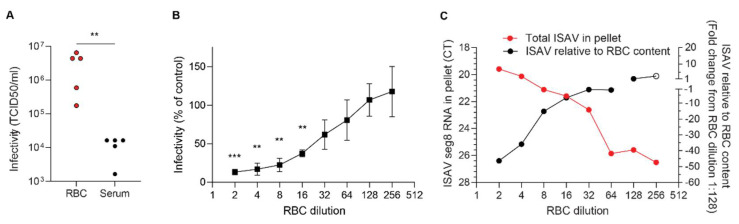
Erythrocytes sequester infective ISAV particles and inhibit serial infection of cultured cells. (**A**) Density gradient-purified erythrocytes (RBC) and plasma samples were harvested from ISAV-infected fish (13 d.p.i. in the 2020 trial) and inoculated on ASK cells. Data points represent samples from individual fish. ** *p* = 0.079, Mann–Whitney U. (**B**) Serially diluted RBC from healthy fish were added to infected ASK cells 24 h p.i. and incubated another 24 h before supernatants were transferred to new ASK cells. Infection of ASK cells 72 h.p.i. was measured by immunostaining for ISAV NP and automated quantification of the number of infected cells (Spectramax i3x plate reader, minimax 300 Imaging Cytometer module, Molecular devices, CA, USA). The infectivity graphs show medians (dots) and 95% confidence intervals (bars), representative of two independent experiments. *** *p* < 0.001, ** *p* < 0.01, one sample t test. (**C**) Serially diluted RBC from healthy fish were incubated with ISAV. The total levels of ISAV RNA associated with the diminishing RBC pellets were measured by qPCR (red, left *y*-axis). ISAV RNA relative to the RBC content in the pellet was calculated by the ∆∆CT method, using ELF1α as reference gene and the highest dilution of RBC with ELF1α CT < 35 (considered the reliable limit of detection) as reference sample (black, right *y*-axis, open circle indicates ELF1α CT > 35). Data show the means of technical duplicates representative of two independent experiments.

In support of with our qPCR findings, inoculation of ASK cells with plasma and erythrocytes from infected fish suggested that the number of infectious particles in erythrocytes far exceeded that in plasma (Figure 7A). In this experiment, 10 μL plasma or 10 μL density gradient-purified erythrocytes were added to 500 μL culture medium, stored at −80 °C, and titred as described in Materials and Methods. Infective titres of blood cell fractions were high, with levels similar to crude virus supernatants propagated in ASK cell cultures. Our findings show that erythrocyte-bound ISAV particles remain infective. We next added erythrocytes to infected ASK cultures 24 h.p.i. and transferred the supernatants to uninfected ASK cells. We found that the presence of erythrocytes inhibited secondary infection in a dose-dependent manner (Figure 7B). The reduced infectivity of supernatants was mirrored by a rise in total erythrocyte-associated ISAV RNA, reflecting the number of erythrocytes in the assay (Figure 7C, red). When the erythrocyte content in the pellet fell, ISAV RNA relative to cellular RNA increased (Figure 7C, black), suggesting that the erythrocyte capacity to bind ISAV was not saturated at high erythrocyte concentrations. Our findings suggest that erythrocytes can act as decoys, at least in the in vitro setting.

## 4. Discussion

We here show that viraemia, including coating of erythrocytes with ISAV particles, is a consistent feature of ISA. Because the erythrocyte is the most numerous cell type in the body and travels to every tissue, it is relevant to ask what this means for the course of infection and the development of disease.

### 4.1. Erythrocytes Do Not Play a Significant Role in ISAV Replication

The first main implication of our findings is that circulating erythrocytes do not appear to play a significant role in ISAV replication, despite occasional expression of viral proteins in a small cellular subset. Viruses replicate by infecting permissive host cells, hijacking the cellular machinery to reproduce themselves. Different cell types permit replication of specific viruses, depending on properties of both the cell and the virus. For enveloped viruses, cellular entry requires the presence of a viral receptor on the cell surface together with the ability to fuse with the cell membrane and release viral contents to the cytoplasm [32]. The ISAV receptor is predominantly found on erythrocytes and endothelial cells in Atlantic salmon tissues [8]. Endothelial cells express high levels of ISAV proteins, as demonstrated by immunohistochemistry [8]. Moreover, ultrastructural studies show that ISAV particles bud from their luminal surface [8,11], supporting that endothelial cells are permissive to ISAV infection and that most ISAV particles observed in blood smears originate from viral replication in the endothelium.

In contrast, ISAV protein expression has never before been demonstrated in erythrocytes of infected fish. We do not know if the observed protein expression in the 2018 trial was associated with completion of the infectious cycle and the release of new viral particles. For example, chicken erythrocytes produce ample viral proteins after in vitro inoculation with avian influenza virus, but fail to generate new infective progeny [33]. Nevertheless, the strong expression of viral proteins in a limited subset of cells demonstrate that ISAV, at least under some circumstances, can be internalised in circulating erythrocytes of infected Atlantic salmon and engage these cells in transcribing and translating viral gene products.

Importantly, several lines of evidence suggest that such ISAV protein-expression by erythrocytes is a rare event that is not required for the development of anaemia and disease: First, erythrocyte expression of ISAV proteins was only observed in one of the trials included in our study. Second, even in that trial, the fraction of erythrocytes expressing ISAV proteins was less than 5%, and the relative levels of viral transcription in full blood were no different from that in erythrocytes in the less severe trial. In contrast, considerably higher ISAV transcription levels were detected in the heart and kidney in the less severe trial. Third, in vitro inoculation of salmon erythrocytes with ISAV did not result in viral replication. Finally, we observed that erythrocyte-associated ISAV remained infective, suggesting that at least a proportion of virus particles associated with circulating erythrocytes fail to fuse with the erythrocyte membrane.

Our findings imply that most circulating Atlantic salmon erythrocytes are non-permissive to ISAV. Similar to our observations for ISAV, rainbow trout erythrocytes appear to be non-permissive to infectious pancreatic necrosis virus [34] and viral haemorrhagic septicaemia virus [35]. However, this is not the case for all viruses: in the active stage of piscine orthoreovirus infection, circulating Atlantic salmon erythrocytes appear to support both viral transcription and protein expression [36]. Moreover, Atlantic salmon erythrocytes inoculated with piscine orthoreovirus ex vivo support the generation of new infective viral particles [37].

Many factors could contribute to the variation in teleost erythrocyte permissiveness to different viruses, including the efficiency of viral entry, evasion of intrinsic antiviral responses, complexity of the virus particle, and factors associated with engaging the Atlantic salmon erythrocyte transcriptional and translational machinery. For example, piscine orthoreovirus has a fully cytoplasmic replication cycle, while ISAV, like influenza viruses and other members of the *Orthomyxoviridae* family, requires a stage of nuclear transcription [38]. On the other hand, human parvovirus B12, a single-stranded DNA virus that also requires nuclear transcription, replicates extensively in nucleated human erythroid precursors, showing that nuclear viral replication is possible in the erythroid lineage, at least at early stages of differentiation [39,40].

Virus-specific differences may furthermore be relevant to explain the apparent contradiction between the lack of ISAV replication in cultured erythrocytes in our study and a previous study that reported replication of the North-American ISAV isolate NBISA01 in agglutinated blood [23]. European and North-American ISAV genogroups are genetically distinct [41], and it is possible that differences in their genetic make-up could account for this discrepancy. However, because no purification step was reported when preparing erythrocytes for the haemagglutination reaction in the previous study, it is also possible that other cell types than erythrocytes may have contributed to the observed viral replication.

The erythrocyte differentiation stage should also be considered in the interpretation of our findings. Even in teleost fish, where erythrocytes retain their nuclei throughout their life span, young erythrocytes appear to have a more active transcriptional and translational machinery than older subsets [42] and may as such be better suited for viral replication. For example, in the persistent stages of piscine orthoreovirus infection when little viral protein is associated with circulating erythrocytes, the virus replicates in early erythroid precursors in the kidney [43]. Because of their rare occurrence, it was not possible to determine if the ISAV protein-expressing erythrocytes detected in our study represented a distinct erythrocyte subset. However, the erythrocyte viral protein expression was limited to the trial with the most severely anaemic fish, suggesting high levels of immature erythrocytes in the circulation. Moreover, we also observed ISAV protein-expression in rounded cells that could be consistent with an immature erythroid phenotype.

### 4.2. Erythrocytes Sequester Active ISAV Particles during Infection

While the contribution of erythrocytes to ISAV replication appears to be negligible, the extensive binding of infective ISAV particles to circulating erythrocytes suggests a potential to modulate infection dynamics and pathogenesis in ISA. Although it should be kept in mind that a direct comparison of plasma and erythrocytes, two very different biological substances, has some limitations, our results strongly suggest that most ISAV RNA in blood of infected Atlantic salmon was associated with the erythrocyte fraction, rather than free in the plasma. In support of most ISAV being found in the cellular fraction of blood, inoculation of permissive cells with erythrocytes from infected fish resulted in productive infection of cultured cells. Much lower infective titres were detected in plasma.

It is very relevant to ask how this association between infective viral particles and erythrocytes influences the distribution of ISAV during infection. For example, does it influence the circulation half-life of viral particles or the potential for viral dissemination? Binding to erythrocytes has been studied in other viral infections. In human patients with chronic HIV, infective HIV-1 particles remained associated with erythrocytes even though virus levels in plasma were suppressed to undetectable levels [44]. Moreover, the association with erythrocytes appeared to promote HIV-1 trans-infection of peripheral blood mononuclear cells [45]. For adenovirus of the Ad5 serotype, binding to erythrocyte coxsackievirus and adenovirus receptor prolonged the circulation half-life of the virus in mice, but reduced the extent of hepatic infection [46]. Erythrocyte binding also modulates the availability of other circulating molecules. For example, plasma levels of inflammatory chemokines are buffered by their binding to erythrocyte receptors without signalling functions [47,48].

With regards to infectivity, we found that erythrocytes sequestered infective viral particles produced in ASK cells and limited serial infection. It is not yet clear how these findings translate to the situation in infected Atlantic salmon. After the initial replication phase in mucosal surface epithelium, infection with ISAV results in a disseminated pan-endothelial infection pattern [8,10]. The exact route by which epithelial-released ISAV infects endothelial cells has not yet been determined, but sialic acids are mainly expressed on the luminal surface of endothelial cells [49]. It is therefore reasonable to assume that the infection of endothelial cells in distant internal organs involves dissemination of blood-borne virus particles. While the peak of viraemia occurs at the same time as or after the peak of viral transcription in heart and kidney [12,24], we detected viral proteins on erythrocytes as early as 4 d.p.i. in the most severe trial. Whether the binding of ISAV to erythrocytes limits the ability to infect distant sites, as in our cell model system, or if it promotes infection, by protecting the virus from antiviral factors in serum, for example, currently remains an open question.

We observed that the ISAV-coating of erythrocytes preceded the onset of anaemia. Accordingly, one should also consider how the binding of virus particles influences the fate of erythrocytes. The presence of viral proteins on the erythrocyte surface could result in targeting by complement, immune complexes, and/or scavenger receptors, thus augmenting the rate of erythrocyte removal from the circulation. This assumption is supported by the observation of regenerative anaemia [25] and splenic haemophagocytosis in ISA [8]. We also observed a mild increase in erythrocyte osmotic fragility in ISAV-infected fish, although less pronounced than in a previous report examining blood samples obtained from Norwegian ISA outbreaks in the late 1980s [28]. The osmotic fragility assay measures the cellular ability to take up water and swell without bursting, in response to reduced extracellular osmotic pressure. The osmotic fragility may be influenced by a range of factors, including erythrocyte swelling, loss of membrane components, or a reduction in the erythrocyte plasma membrane integrity. We did not address if the observed increase in osmotic fragility resulted from direct viral interactions with the plasma membrane. Hence, our data do not exclude that other factors, including secreted stress hormones or other signalling molecules, could be involved. Another interesting question for future studies is whether the scavenging of ISAV-coated erythrocytes could have potential to modulate the immunological response, considering that antigen-coating of mouse erythrocytes appears to induce broad immunological tolerance [50].

### 4.3. The Susceptibility to Infectious Salmon Anaemia Depends on Many Factors

The development of disease and mortality was very different in the two trials included in this study, despite viral kinetics suggesting that all exposed fish were infected at the first exposure. The trials were performed in two different locations, using fish from the same source, but of different year classes and reared in different locations. Hence, we were not surprised that disease outcome, as measured by cumulative mortality, was different. Practical experience in our research group from a large number of ISAV infection trials over the last three decades suggests that a number of different factors, some of which remain poorly understood, affect ISA disease outcome [personal observation, Knut Falk]. These factors are only in part described in the literature and include the genetic background of the fish [51,52]; the virus isolate [2,12,53]; the route and dose of infection [24,54]; trial environmental factors such as water temperature, water quality, and experimental facility; factors associated with the upbringing of the fish from the larval stage; and the time of year. The two trials described here infected the fish with the same ISAV isolate administered by the same route and at similar water temperatures. The most obvious trial design differences were that the fish were from different year classes and reared in different locations, a six-fold difference in infective dose, the different experimental facilities, and that one trial was performed in salt and the other in fresh water. Note that previous trials with the Glesvaer/2/90 isolate have resulted in 100% mortality at the same infective dose as in the less severe experiment [2] and in both salt and fresh water [2,12]. It is therefore tempting to speculate that other factors, perhaps associated with genetics or upbringing of the fish, have contributed to the observed difference in cumulative mortality. In conclusion, our observations underscore the need for better knowledge of factors contributing to the development of disease and death after ISAV infection.

### 4.4. ISAV Binding to Erythrocyte Membranes

Erythrocyte membrane proteins appear to be well conserved across species, and the most prominent ISAV-binding protein band in Atlantic salmon erythrocyte membrane fractions resembled that of the human band 3 anion transporter [55]. This band was observed at the level of the 80 kDa molecular marker. Note that the migration of heavily glycosylated proteins is strongly influenced by their charge, and molecular weights cannot be reliably estimated from migration patterns. In contrast to humans, where the most prominent PAS-staining band (PAS-1) is located below band 3, we detected strong PAS-staining at the same level as the band 3-like band. This is similar to previous observations in carp and trout [27,55]. Of two other clearly PAS-positive bands between the 50 and 60 kDa markers, only the upper one bound ISAV, most likely reflecting the presence of 4-*O*-acetyled sialic acid [9]. We also detected two lower ISAV-binding bands with no obvious associated PAS staining. Nevertheless, 4-*O*-acetylated sialic acid remains the only known receptor for ISAV [9], and de-acetylation by saponification obliterated all ISAV-binding to the membrane. We propose that the two lowest ISAV-binding bands most likely contain glycoproteins, but at levels below the detection limit of the glycoprotein staining assay. Teleost membrane glycoproteins are poorly characterised, and it is not known whether they oligomerise, but based on knowledge from human erythrocyte glycoproteins [55], some of the ISAV-binding bands could represent different –meric states of the same glycoprotein.

## 5. Conclusions

Our study sheds light on several aspects of ISAV-erythrocyte interactions: first, by documenting that coating of circulating erythrocytes with infective viral particles is a consistent feature of ISA; second, by suggesting that erythrocytes do not significantly contribute to ISAV replication; and third, by discussing possible mechanisms by which the documented binding of ISAV to circulating erythrocytes could modulate ISAV infection dynamics and ISA pathogenesis.

## Data Availability

Data is contained within the article. Original uncropped images of blots and micrographs have been uploaded as supporting information. In addition, .czi files can be obtained upon request.

## Supplementary Materials

The following are available online at https://www.mdpi.com/article/10.3390/v14020310/s1, Table S1: Primers/probe sets. Table S2: Synthetic DNA used for standard curve (gBlocks).
