# Supplementary material for: Salmon Erythrocytes Sequester Active Virus Particles in Infectious Salmon Anaemia
Source: Viruses. 2022 Feb 2;14(2):310. doi: 10.3390/v14020310 (PMC8879071; doi:10.3390/v14020310)
Supplement: Supplementary file 1 [file viruses-14-00310-s001.zip › viruses-1501741-supplementary.pdf]

viruses

MDPI

Supplementary Materials and Methods

Table S1. Primer/probe sets.

Target		Primer/Probe Sets
Elongation factor 1α[1*] (ELF-1α)	Forward	GGCTGGTTCAAGGGATGGA
	Reverse	CAGAGTCACACCATTGGCGTTA
	Probe	FAM-TCGAGCGTAAGGATG-NFQ
ELF-1α[1]	Forward	CCCCTCCAGGACGTTTACAAA
	Reverse	CACACGGCCCA-CAGGTACA
	Probe	HEX-CTCTCTCATTGTGATCCC-MGB/NFQ
IFNa3-like, transcript variants X1 and X2[1]	Forward	TGCAGTATGCAGAGCGTGTG
	Reverse	TCTCCTCCCATCTGGTCCAG
	Probe	*NA*
Mx, transcript variant X1[1]	Forward	GGTGATAGGGGACCAGAGT
	Reverse	CTCCTCACGGTCTTGGTAGC
	Probe	*NA*
Seg8[2]	Forward	CGAAAGCCCTGGAAACTTTAGA
	Reverse	GATGCCGGAAGTCGATGAACT
	Probe	FAM-AAGGCCATCGTCGCT-MGB/NFQ
Seg8[2]	Forward	CTACACAGCAGGATGCAGATGT
	Reverse	CAGGATGCCGGAAGTCGAT
	Probe	FAM-CATCGTCGCTGCAGTTC-MGB/NFQ
Seg7 ORF1[2]	Forward	CAGGGTTGTATCCATGGTTGAAATG
	Reverse	GTCCAGCCCTAAGCTCAACTC
	Probe	FAM-GGGATCACAATGAGAGA-MGB/NFQ
Seg7 ORF2[2]	Forward	ACGGAAAAGACAAGGTGGCTT
	Reverse	CCATCCTCCCCAGGAACCT
	Probe	FAM-TTCCTGTCGGGCTCA-MGB/NFQ

[1] Atlantic salmon, [2] ISAV, NA = not applicable, * primers also used without probes in SYBR green assay.

Table S2. Synthetic DNA used for standard curve (gBlocks).

Targets	gBlock Sequence (Primer/Probe Targeted Sequences Underlined)
ELF-1α[1]-Seg8[2]	CATGCTGGAGGCTAGCGCCAACATGGGCTGGTTCAAGGGATGGAAGGTCGAGCGTAAGGATGGTAACG<br>CCAATGGTGTGACTCTGCTGGAAGCCCTGGACGATGCAGATGTATGCTCTAGGAGCGAGTTCGAAAGCC<br>CTGGAAACTTTAGAAAAGGCCATCGTCGCTGCAGTTCATCGACTTCCGGCATCCTGCTCGACAGAGAAG<br>ATGGTGCCAGGGTTGTATCCATGGTTGAAATGGACAGAGACGGCGTATCATTCATCTACGAGAAGCCTA<br>GCATCTACCATAGTGATGGGTGCACTGGGACAGCATCGAGGGTCTGGAGACGGGATCACAATGAGAGA<br>GCTGGAGTTGAGCTTAGGGCTGGACTTCACTTCAGAA
Seg7 ORF1[2]-Seg7 ORF2[2]-Seg8[2]	AGCTAAGATTCTCCTTCTACAATGGATTTCACCAAAGTGTATGGTGTGCTGGTTGACCAACTAAAACTTC<br>ACGGAAAAGACAAGGTGGCTTCTTTCCTGTCGGGCTCAAAGGTTCCTGGGGAGGATGGTATCTCAAGTA<br>CGTCAGGTATGCTGGACCTCTTGAAGGTGCTACACAGCAGGATGCAGATGTATGCTCTAGGAGCGAGTT<br>CGAAAGCCCTGGAAACTTTAGAAAAGGCCATCGTCGCTGCAGTTCATCGACTTCCGGCATCCTGCTCGA<br>CAGAGAAGATGGTGCCAGGGTTGTATCCATGGTTGAAATGGACAGAGACGGCGTATCATTCATCTACGA<br>GAAGCCTAGCATCTACCATAGTGATGGGTGCACTGGGACAGCATCGAGGGTCTGGAGACGGGATCACA<br>ATGAGAGAGCTGGAGTTGAGCTTAGGGCTGGACTTCACTTCA(GAA)[3]

[1] Atlantic salmon, [2] ISAV, [3] One variant of this gBlock did not include the nucleotides in brackets.
